# Metagenomic assessment of the global diversity and distribution of bacteria and fungi

**DOI:** 10.1111/1462-2920.15314

**Published:** 2020-12-02

**Authors:** Mohammad Bahram, Tarquin Netherway, Clémence Frioux, Pamela Ferretti, Luis Pedro Coelho, Stefan Geisen, Peer Bork, Falk Hildebrand

**Affiliations:** ^1^ Department of Ecology Swedish University of Agricultural Sciences Uppsala Ulls väg 16, 756 51 Sweden; ^2^ Department of Botany Institute of Ecology and Earth Sciences, University of Tartu Tartu 40 Lai St. Estonia; ^3^ Gut Microbes and Health Quadram Institute Bioscience Norwich, Norfolk UK; ^4^ Digital Biology Earlham Institute Norwich, Norfolk UK; ^5^ Structural and Computational Biology European Molecular Biology Laboratory Heidelberg Germany; ^6^ Department of Terrestrial Ecology Netherlands Institute of Ecology NIOO‐KNAW Wageningen 6708 PB The Netherlands; ^7^ Max Delbrück Centre for Molecular Medicine Berlin Germany

## Abstract

Bacteria and fungi are of uttermost importance in determining environmental and host functioning. Despite close interactions between animals, plants, their associated microbiomes, and the environment they inhabit, the distribution and role of bacteria and especially fungi across host and environments as well as the cross‐habitat determinants of their community compositions remain little investigated. Using a uniquely broad global dataset of 13 483 metagenomes, we analysed the microbiome structure and function of 25 host‐associated and environmental habitats, focusing on potential interactions between bacteria and fungi. We found that the metagenomic relative abundance ratio of bacteria‐to‐fungi is a distinctive microbial feature of habitats. Compared with fungi, the cross‐habitat distribution pattern of bacteria was more strongly driven by habitat type. Fungal diversity was depleted in host‐associated communities compared with those in the environment, particularly terrestrial habitats, whereas this diversity pattern was less pronounced for bacteria. The relative gene functional potential of bacteria or fungi reflected their diversity patterns and appeared to depend on a balance between substrate availability and biotic interactions. Alongside helping to identify hotspots and sources of microbial diversity, our study provides support for differences in assembly patterns and processes between bacterial and fungal communities across different habitats.

## Introduction

Bacteria and fungi contribute significantly to global biodiversity and biomass (Bar‐On *et al*., 2018), and are fundamentally important for global ecosystems and host health and functioning. Bacteria provide their hosts with vitamins and cofactors, act as plant‐growth promoting rhizobacteria, and help digest otherwise indigestible fibres and contribute to immunity (Schmidt *et al*., 2018). While some fungi are known as important plant symbionts or comprise some of the most beneficial mutualists and detrimental pathogens (Fisher *et al*., 2012), the role of fungi in non‐plant hosts is less known. In environmental habitats, both bacteria and fungi drive decomposition of organic material and nutrient cycling (Falkowski *et al*., 2008; Berendsen *et al*., 2012). Given that hosts exist within external environments, external host habitats are much more complex in terms of scale, abiotic heterogeneity and C resources, compared with internal host habitats. Thus, environmental microbiomes – and to a lesser extent external host microbiomes – likely experience greater exposure to migrations of different organisms/genes, leading to a greater variety of niches suitable for the establishment of a larger variety of organisms compared with internal host habitats (Pent *et al*., 2017; Küngas *et al*., 2020). Different abiotic factors such as pH, climate and organic matter contents shape the environmental community compositions of bacteria and fungi (Tedersoo *et al*., 2014; Louca *et al*., 2016; Bahram *et al*., 2018; Delgado‐Baquerizo *et al*., 2018). Accumulating evidence hints at the niche specialization of bacteria and fungi, reflected in their global distribution patterns (Frey‐Klett *et al*., 2011; Bahram *et al*., 2018; Crowther *et al*., 2019), suggesting that contrasting mechanisms underlie their community assembly processes. At the same time, there appears to be a significant functional overlap between bacteria and fungi for utilizing resources, hinting at the importance of bacterial–fungal interactions (de Boer, 2017). Yet, a simultaneous synthesis of bacterial and fungal community patterns across a wide array of host and environmental habitats is so far lacking.

We have profoundly increased our understanding of global patterns of both bacterial and fungal communities, but most studies tend to examine either bacterial or fungal communities in isolation, often without determining their associated functions. Given that microbial genes and taxa appear to be exchanged across different host and environmental habitats (Sokol *et al*., 2017; Bahram *et al*., 2018; Hannula *et al*., 2019), it remains an open question whether stochastic processes (geographical proximity, random dispersal), or deterministic processes (similarity in environmental conditions, or biotic interactions) determine microbiome structure across habitats. Aside from other abiotic factors, the composition of microbial communities appears to depend on the prevailing and dominant available source of organic carbon (C) in a given habitat (Hoffmann *et al*., 2013). In general, it is thought that fungi tend to outcompete bacteria in utilizing more complex and varied forms of C, whereas bacteria tend to outcompete fungi for more labile C sources in terrestrial habitats (Boer *et al*., 2005; Žifčáková *et al*., 2017). This is likely facilitated by physiological differences, including contrasting stoichiometry, carbon use efficiencies, enzymatic capabilities, and stress tolerance mechanisms between bacteria and fungi (Lynch and Walsh, 2007; Frey‐Klett *et al*., 2011; Sokol *et al*., 2017; Bahram *et al*., 2018; Deveau *et al*., 2018; Naranjo‐Ortiz and Gabaldón, 2019). Yet, the role of bacteria in the decomposition of more recalcitrant forms of C and the role of fungi in the utilization of more labile C may be underestimated, and both fungi and bacteria may be equally important in overall C decomposition patterns (Strickland and Rousk, 2010; Bugg *et al*., 2011; de Vries and Caruso, 2016; Wilhelm *et al*., 2019). There is also growing evidence that the microbial control of ecosystem processes as well as host health may be mediated by biotic interactions between bacteria and fungi (Mendes *et al*., 2011). These interactions span an antagonistic–mutualistic spectrum to direct predation and parasitism, and range from free‐living interactions to mixed biofilms, and intrahyphal colonization by bacteria (Frey‐Klett *et al*., 2011). Collectively these growing insights into the specific properties of bacteria and fungi and their interactions suggest that habitat types may have distinct bacterial‐fungal (B/F) ratios and alterations to the B/F balance in either direction may have consequences for ecosystem and host functioning, e.g. in C decomposition, and host health (Allison *et al*., 2013; Bahram *et al*., 2018).

Here, by leveraging a global dataset of 13 483 metagenomic samples, we examined the structure and function of fungal and bacterial communities, based on the relative abundance of taxonomic stable rRNA genes and orthologous groups (OGs) respectively (Fig. S1; Table S1). Metagenomics approach allows us to compare fungal and bacterial compositions simultaneously, and in relation to each other (Bahram *et al*., 2018). This may offer advantages over 16S or ITS amplicon sequencing that can only amplify bacteria and fungi, making relative comparisons between them riddled with biases (Handelsman, 2004; Hugenholtz and Tyson, 2008; Grice and Segre, 2011; Tedersoo *et al*., 2015; Quince *et al*., 2017). Our selection of publicly available samples included 25 habitat types, spanning internal and external host (including skin, oral and gastrointestinal of different animal species) and environmental (including soil, water and built) habitats. These habitats differ in environmental variation, scale, biotic interactions and dispersal processes. Therefore, we hypothesized that: (i) there is a greater diversity (Shannon diversity index) and relative abundance of both bacterial and fungal taxa and associated functions in environmental and external host compared with internal host microbiomes, where microbes and their functions are likely to be more specialized, and that (ii) certain host associated microbes are likely to be a subset of the microbiome metacommunity of the environment, i.e. displaying a nested community structure. More specifically, due to greater exposure to environmental microbes, external host habitats more likely harbour a subset of microbes from the environment, compared with internal host habitats. We also hypothesized that: (iii) bacteria show stronger habitat associations compared with fungi, due to the greater effect of deterministic than stochastic processes in shaping bacterial communities; and (iv) the relative abundance and diversity ratios of bacteria to fungi decrease in environments with more complex plant‐derived C resources, i.e. soils, due to the greater affinity of fungi to plants and their associated resources.

## Results and discussion

**Fig 1:**
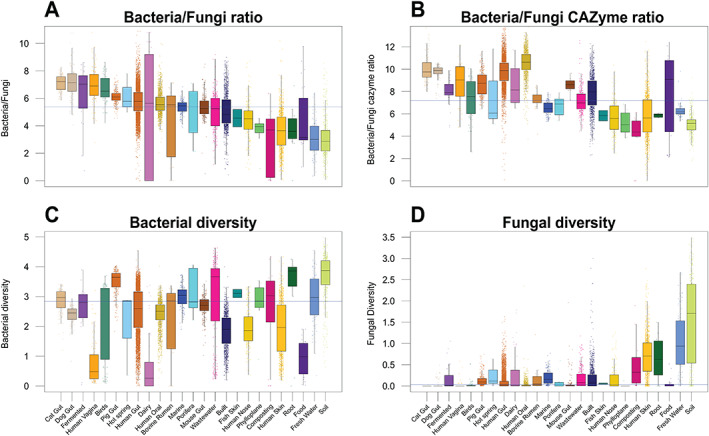
abundance and diversity of bacterial and fungal classes across diverse habitats. A and B. Bacterial/fungal (B/F) rRNA and CAZyme gene ratios. C and D. Bacterial and fungal rRNA gene diversity respectively. These data show that the relative abundance and diversity of fungi is higher in terrestrial and aquatic habitats respectively, whereas bacteria show the highest relative abundance in nutritional habitats possibly because greater available nutrition resources for growth. Blue line specifies the median across all habitats. Diversity was calculated based on Shannon index using the genus level abundance matrix, whereas B/F ratio was calculated based on the abundance of SSU reads assigned to bacteria and fungi.

Not surprisingly, bacteria greatly exceeded fungi numerically across samples, being on average 700‐fold relatively more abundant across all habitats (Fig. 1; Table S1). Both bacteria and fungi, but even more so fungi, displayed higher diversity in terrestrial habitats, particularly in soils and rhizospheres than in marine habitats (Figs. 1, [Link], [Link]). Overall, and partially confirming our first hypothesis, internal host‐associated microbiomes were less diverse than most external host‐associated and environmental microbiomes (Fig. 1C,D), corroborating the findings from a recent global bacterial metabarcoding analysis (Thompson *et al*., 2017).

In addition, in line with our first hypothesis, among host habitats, fungi showed the greatest relative abundance as well as diversity in those with direct external exposure such as human nose, oral and skin (Figs. 1, [Link], [Link]). Such habitats are possibly exposed to a greater influence of external environmental factors and connectivity to other habitats leading to a higher exchange of fungal taxa. These effects are less pronounced for bacteria, likely due to their greater dispersal potential across habitats. In addition, the B/F ratio was greater in habitats with presumably higher nutrient availability and lower C/nutrient ratios, which might reflect the greater metabolic flexibility, carbon‐use‐efficiency, and competitive ability of bacteria compared with fungi in nutrient and soluble C rich environments (Averill and Hawkes, 2016; Bahram *et al*., 2018). For example, we found a strong association between fungi and the herbivore lifestyle in gastrointestinal tract (GI) communities: plant‐fed mice GI and bovine rumen had on average twofold greater fungal relative abundance (mean B/F = 220) compared with omnivores (pig and human GI, mean B/F = 383) and sixfold more compared with carnivores (cat and dog GI, mean B/F = 1305) (Fig. 1). Despite having several orders of magnitude less abundance than bacteria (Fig. 1), fungi may have a disproportionate ability to thrive in spatially and temporally heterogeneous conditions, largely airborne dispersal mechanisms and the production of hyphae (Fig. S7).

**Fig 2:**
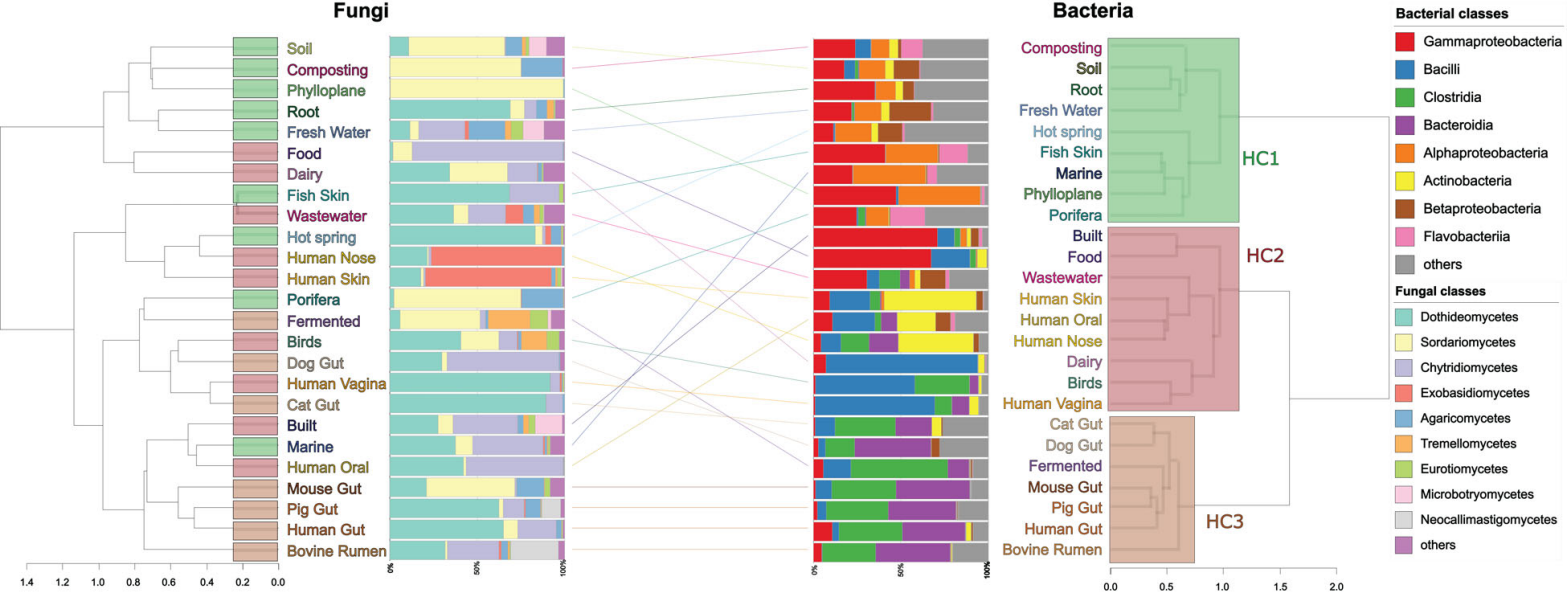
Habitat association of fungi and bacteria. Clustering of microbial habitats based on fungal classes (left) and bacterial classes (right). Between sample Bray–Curtis distance was averaged between samples of biomes, to create a hierachical clustering (ward.d2). Median fungal and bacterial class abundances are shown next to environmental clustering. The three bacterial habitat clusters (HCs) roughly correspond to different balances of the B:F ratio. HC1 (environmental) seems to be driven by the presence and dominance of fungi and complex sources of C. In this cluster, bacteria could rely more on fungal‐derived C as well as symbioses and hence more secondary metabolites for enhanced interactions, and these bacterial‐fungal interactions drive the bacterial composition and diversity of environmental habitats. HC2 (external host and human influenced environmental habitats) seems to represent ecotones, transitions between more isolated habitats, whereas HC3 (anaerobic isolated habitat like guts) perhaps represents the most specialized habitat which favours bacteria due to more homeostatic conditions (pH, temperature and moisture). Fungi show more diffuse clustering which might be due to their large reliance on passive dispersal (largely airborne), strong affinity to plants and plant‐derived C, and less habitat specificity. The top bar plot shows the out of‐bag variance explained for each model with the dependent variables on the x‐axis.

To determine whether similar habitats in terms of conditions and connectivity shape microbial communities, and to test our hypothesis as to whether bacteria have greater habitat associations compared with fungi and to explore the diversity patterns of different habitat groupings, we performed a hierarchical clustering of habitats based on the composition of bacterial communities. The results revealed three major microbial community clusters (cf. habitat clusters, HC; Fig. 2), that were dominated by environmental (HC1), host associated external (HC2) and host associated internal (HC3) habitats. HC1 included relatively more diverse bacterial communities, and Alpha, Beta and Deltaproteobacteria (Fig. 2). HC2 had increased Actinobacteria and Gammaproteobacteria, whereas HC3 was dominated by Clostridia and Bacteroidetes (Fig. 2).

**Fig 3:**
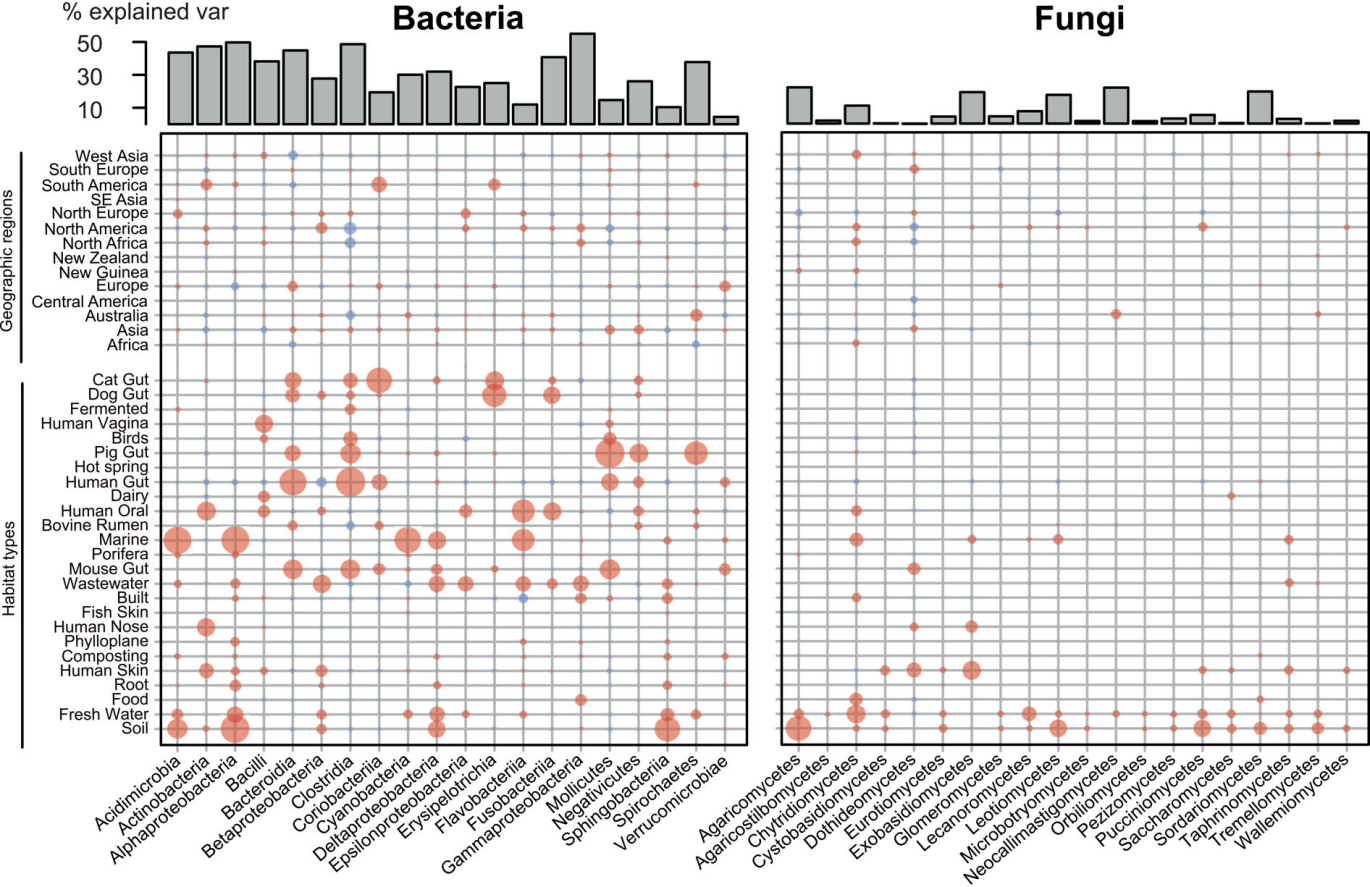
Bacteria show greater habitat association than fungi across diverse habitats. Heatmap showing habitat and geographic association of the top 20 most abundant bacterial and fungal classes that most strongly correlated with habitat types. The size of circles corresponds to the variable importance (percentage of mean decrease accuracy estimated based on out‐of‐bag‐CV); blue and red depict negative and positive Spearman correlations respectively.

**Fig 4:**
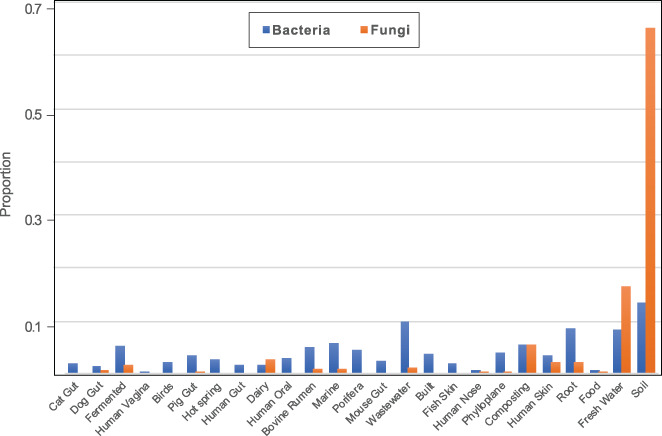
Indicator fungal and bacterial genera across various host and environmental habitats. Bars show the fraction of fungal or bacterial genera significantly associated (FDR < 0.3) with each habitat based on Species indicator analysis (to total number of genera, i.e. 1658 and 3750 for fungi and bacteria respectively). Only genera with more than 10 reads were used in the analysis.

We found support for our second hypothesis that communities of host‐associated microbes are a subset of those in environmental habitats. Notably, dominant fungal, but not bacterial genera, present in the gut were a subset of external host habitats, which were in turn a subset of environmental habitats (Fig. S8). In addition, in line with our third hypothesis, bacteria showed stronger habitat associations and distinctive communities, compared with fungi (Figs. 2, 3, Table S3), perhaps because of their greater environmental associations, comparatively longer evolutionary history and/or greater genomic plasticity (Frey‐Klett *et al*., 2011; Naranjo‐Ortiz and Gabaldón, 2019). This was further supported by analysing habitat‐specific genera, which revealed that 124 fungal genera representing 7.5% of all fungal genera were significantly associated with a specific habitat, compared with 36.3% in the case of 1360 bacterial genera. However, compared with fungi, the proportion of indicator bacterial genera was less contrasting between habitats, yet it was greater in environmental habitats (Fig. 4).

Overall, the clustering of bacterial communities appeared to depend more on habitat conditions, whereas for fungi it appeared to depend on either the interplay between habitat conditions, spatial dispersion and transfer between proximal environments or none of these (Figs. 2, 3, 4). For example, fungal communities in human gut samples clustered with those from the built habitat, so as those from dairy with bovine rumen and human skin. Although this is not direct evidence of a frequent transfer of fungi between these habitats, and we cannot distinguish between transient and non‐transient fungi in specific habitats, our results suggest that the interaction of habitat specificity and spatial proximity could affect fungal community composition across diverse habitats, as shown for dispersal as a main driver underlying stochastic community assembly of soil fungi (Bahram *et al*., 2018), possibly reflecting the reliance of many fungi on airborne dispersal via spores (Huang and Hull, 2017).

The relatively higher abundance of fungi in terrestrial and particularly soil habitats, in line with our fourth hypothesis, may be related to the major diversification events of fungi being tightly linked to those of plants. The most diverse fungal lineages have affinities for symbiotic, pathogenic and saprotrophic interactions with plants and their resources (Lutzoni *et al*., 2018; Tedersoo *et al*., 2018). We suggest that this facilitates the formation of a stable and complex soil fungal communities, whereas stochastic and dispersal‐related processes may have stronger impact on fungal communities in other habitats. This hypothesis is further strengthened by the observation that most of the habitat specific fungal genera were associated with soil, including multiple indicator genera from Agaricomycetes (Fig. 4, Table S3), which is composed mostly of litter decomposing and plant‐associated fungi that thrive in the presence of plant hosts (Lutzoni *et al*., 2018). Archaeorhizomycetes, a recently discovered fungal class (Rosling *et al*., 2011), was also almost exclusively found in soil samples. Several Ascomycetes such as *Penicillium* and *Mycosphaerella* were among indicator genera for soil and rhizosphere habitats, known soil saprotrophs and plant pathogens respectively (Crous *et al*., 2009; Diao *et al*., 2019). Thus, fungi may possess a broad range of C‐cycling enzymes in soils, perhaps due to diverse substrates provided by plants and plant–fungal symbioses.

Accordingly, compared with saprotrophic bacteria, saprotrophic fungi have developed higher and wider C/nutrient stoichiometries, greater C demands, higher carbon‐use‐efficiency with higher C/N resource ratios, and extracellular enzymatic specialization in degrading plant‐derived C under aerobic conditions (Keiblinger *et al*., 2010; Tedersoo *et al*., 2014). This tight association with plants may provide fungi greater access to C in soils. In line with this, environmental habitats, especially soils, showed the highest relative abundance of carbohydrate‐active enzyme genes (CAZymes), which are involved in the construction and breakdown of complex carbohydrates, in fungi relative to bacteria (Fig. 1). The B/F ratio was negatively correlated to the relative abundance and diversity of fungal CAZymes (*r* = −0.449, *P* < 10^−15^, *r* = −0.419, *P* < 10^−15^ respectively), and to lesser extent to those of bacteria (*r* = −0.109, *P* < 10^−15^; *r* = −0.207, *P* < 10^−15^ respectively). From specific CAZymes, those related to degradation of lignin showed the strongest correlation to the B/F rRNA gene ratio (Table S2). The more ubiquitous symbiotic interaction between plants and fungi may enhance fungal fitness in relation to bacteria in plant dominated habitats such as soils (Bahram *et al*., 2020).

There are a number of other potential factors driving interactions as well as – supporting or opposing – habitat associations between fungi and bacteria. Filamentous fungi are especially better suited to deal with more heterogeneous habitats and conditions with resource inequality due to their ability to expand and move towards nutrient rich patches and transfer nutrients through hyphae (Whiteside *et al*., 2019). By contrast, bacteria appear to be more sensitive to pH and nutrient availability than fungi (Bahram *et al*., 2018). As reflected in the HC1 habitat cluster (Fig. 2), these are habitats in which fungi tend to reach their greatest diversity, yet bacteria also thrive here. The mostly hyphal forming fungi in these habitats can act as dispersal vectors (Deveau *et al*., 2018), provide and connect high quality nutrient resources, and alter localized pH levels, thus providing a mechanism for enhanced bacterial activity, which is especially important under stressful conditions (Worrich *et al*., 2017). This, together with diverse exchange of metabolites between bacteria and fungi, may be a driver of the composition and diversity of bacterial communities. In some circumstances fungi may even facilitate certain bacteria that are normally considered slow colonizers and poor competitors, enabling them to become locally abundant in symbiosis (Frey‐Klett *et al*., 2011; Phelan *et al*., 2012; Deveau *et al*., 2018). While fungi are also influenced by these associations, both beneficially and antagonistically through bacterial products, their associations with plants and plant‐derived C is perhaps a stronger factor in shaping their community composition, which is reflected in the relatively lower B/F ratios and higher abundance of fungal CAZymes in environmental habitats, particularly soils (plant dominated habitats) (Figs. 1, S9). Such habitats have a greater exposure to complex C forms and fungal airborne spore dispersal, that would increase the chances of such fungi meeting their preferred substrates (Huang and Hull, 2017).

## Conclusions

Our results hint towards the strong role of environmental filtering in structuring cross‐habitat bacterial but not fungal communities. The overall diversity and abundance patterns of bacterial and fungal taxa and associated functional genes reflect their mostly contrasting C acquisition strategies, dispersal strategies, morphologies, alongside their subsequent coevolution via complex biotic interactions with each other and macro‐organisms under highly variable abiotic conditions. We suggest that future studies simultaneously investigate both bacterial and fungal communities, as well as their functional properties, as their interactions and domain‐specific properties may be a strong factor determining the response of ecosystems to environmental change.

## Experimental procedures

### Raw read processing

In total, 31 287 samples classified as metagenomic from 405 projects were downloaded from the European Bioinformatics Institute (EMBL‐EBI) as of 2018/06/19, using customized scripts available at the fetch‐data/ directory of the Supplemental Software package of Coelho *et al*. (in revision). To confirm the annotation of samples as either metagenomics, the abundance of NOGs reads was considered, i.e. samples with very low NOG abundance relative to the total read were considered as metabarcoding regardless of the information provided by submitters. The basic filtering, functional and phylogenetic profiling are an adapted protocol of the methods used in (Bahram *et al*., 2018). In brief, metagenomic and amplicon sequencing reads obtained from public sources were quality‐filtered, if the observed accumulated error exceeded 2.5 with a probability of ≥ 0.01, or > 1 ambiguous position or a homonucleotide run > 15 bp was present. Reads were trimmed if base quality dropped below 20 in a window of 15 bases at the 3′ end, or if the accumulated error exceeded 1 using the sdm read filtering software (Hildebrand *et al*., 2014). Furthermore, all reads shorter than 70% of the maximum expected read length (per sample) were removed. In total, 697 billion total reads were analysed of which 286 + 253 billion (read pair one and two respectively) passed quality filtering (Table S1). After quality filtering and exclusion of samples from underrepresented habitats and with short reads, we analysed 13 483 samples belonging to 25 habitats. The habitats human gut (*n* = 7732) and human oral (*n* = 1586) were the most often represented in our dataset, whereas hot springs were poorly represented (*n* = 4). Most samples originated from North America (24.0%) and Asia (37.0%). Several geographic regions were not represented in our datasets, including Pacific Ocean, North Sea, Arctic, Atlantic Ocean (Table S1).

### Taxonomic annotations

We used a miTag approach implemented in MATAFILER (Hildebrand *et al*., 2019) to determine bacterial and fungal community composition from metagenome sequence data at the higher taxonomic level, detailed in (Bahram *et al*., 2018). Briefly, SortMeRNA (Kopylova *et al*., 2012) was used to extract potential rRNA genes against the SILVA database version 128 (Quast *et al*., 2012). For this, we used SSU rRNA gene for taxonomic identification, which is a universal marker for both prokaryotes and eukaryotes. Reads approximately matching these databases with e‐values < 10^−4^ were further filtered with custom Perl and C++ scripts, using FLASH to attempt merging all matched read pairs. In case read pairs could not be merged, single reads were interleaved such that the second read pair was reverse complemented and then sequentially added to the first read. Lambda (Hauswedell *et al*., 2014) was used to fine‐match candidate interleaved or merged reads to Silva 128 database. The lowest common ancestor (LCA) algorithm adapted from LotuS (Hildebrand *et al*., 2014) was used to determine the identity of filtered reads based on Lambda matches. This included a filtering step, where queries were only assigned to phyla and classes if they had at least 88% and 91% similarity to the best database hit respectively, thresholds adopted from literature (Yarza *et al*., 2014). We normalized each taxon by dividing by the total number of reads per sample to account for uneven sequencing depth across samples. Functional annotation of fungal taxa was done using FunGuild database (Nguyen *et al*., 2016).

### Functional annotations

We used a direct a Blast search approach to estimate the functional gene composition of each sample. The quality‐filtered reads pairs were first merged using FLASH (Magoč and Salzberg, 2011). In cases were read pairs were not available, singleton reads were used instead. These merged, unmerged and singleton reads were mapped against functional reference sequence databases using DIAMOND 0.9.4 (Buchfink *et al*., 2014) in blastx mode with the ‘‐k 5 ‐e 1e‐4 – sensitive’ parameters. If two unmerged query reads mapped to the same target, the mapping scores were combined to avoid double counting dependent reads. In such cases, the hit scores were combined by selecting the lower of the two e‐values and the sum of the bit scores from the two hits. Based on the highest bit score, longest alignment length and highest percentage identity to the subject sequence the best hit for a given query was selected. Finally, reads with an alignment identity < 50% and matching with an e‐value >1 e‐9 were excluded.

For functional annotations, we used *in silico* annotations of metagenomic reads based on a curated database of the orthologous gene family resource eggNOG 4.5 (Huerta‐Cepas *et al*., 2015). eggNOG taxonomic information was used, as reads were mapped competitively against all domains and assigned into prokaryotic and eukaryotic origin, based on the best bit score in the alignment and the taxonomic annotation provided with the database at domain level. In order to estimate the potential of microbes in using C resources, we decided to use the extended orthology represented in eggnog, combined with the precise experimentally validated and functional specific carbohydrate‐active enzyme (CAZyme) annotations (Cantarel *et al*., 2009). For this, we mapped all eggNOG 4.5 amino acid sequences onto the latest CAZy (2019) database (http://bcb.unl.edu/dbCAN2/download/) using DIAMOND. Only high‐quality hits (%id > 90, eval < 1e‐20, > 80% subject coverage) were accepted to ensure that only valid ‘seeds’ were retrieved. From these, the eggnog numbers corresponding to CAZymes based on homology searches to the CAZyme database were retrieved. We used our previously derived eggNOG abundance matrix to obtain a CAZyme profile per sample.

All functional abundance matrices were normalized by the total number of reads used for mapping in the statistical analysis, unless mentioned otherwise (e.g. rarefied in the case of diversity analysis, see below). This normalization was chosen as it considers differences in library size, since unmapped (that is functionally unclassified) reads are included. It is important to note that functional and taxonomic abundance estimates represent relative proportions of represented categories, because of biases of sequencing technologies in capturing every molecule in samples. This requires, as we have done, to choose statistical tests that do not assume absolute measurements, and centres analysis of this type on comparisons across the set of samples.

### Data analysis

Of note, 13 452 samples were categorized into 25 habitats (after removing potential 16S amplicon sequencing runs and studies misclassified in EBI) using the annotation retrieved from submitters with some modifications; for example, soil and rhizosphere samples were combined as soil (Table S1). For analysing fungal and bacterial diversity, the differences in sequencing depth was accounted for by partial linear regression with diversity and sequence abundance as response and predictor respectively, as used previously in (Tedersoo *et al*., 2014). For analysing relative abundances of genes and taxa, the data were normalized by total sum of metagenomics reads per sample. For analysing taxa and gene compositions, abundance data were normalized using Hellinger transformation in *vegan* (Oksanen *et al*., 2007) of R (R Core Team 2015). The B/F ratio was calculated based on the abundance of SSU reads assigned to bacteria and fungi. Diversity (Shannon Index) was calculated and normalized by the total rRNA gene abundance per sample. To examine diversity, we relied on the diversity of genera, to minimize the mis‐assignments at lower taxonomic level inherent to short reads. Based on our previous study (Bahram *et al*., 2018), we found a strong correlation based on genus and OTU diversities in soils (*r* > 0.8). To test discrimination of the relative abundance of different taxa or functions across habitats, permutational multivariate analysis of variance followed by a generalized canonical discriminant analysis was performed using the candisc package (Friendly *et al*., 2017). To test the associations of taxa, we used a sparse partial least squares analysis, as implemented in the *mixOmics* package (Rohart *et al*., 2017).

To cluster habitats based on their fungal or bacterial composition, miTag tables were filtered for either bacterial or fungal taxa (class for fungi, families for bacteria). These tables were normalized by sum, to obtain a relative proportion of fungi or bacteria only within each sample, filtering taxa with < 1e‐5 fractional abundances on average. To obtain scaled Hellinger matrices, we used the sqrt transformed relative abundances and calculated Bray–Curtis distances among samples. To calculate the distances between single habitats, we calculated the mean distance between the respective habitats, for all combinations of habitat pairs. These were then hierarchically clustered using a ward.D agglomerative clustering as implemented in *hclust* and visualized using the tanglegram function from *dendextend* (Galili, 2015), combined with itol's visualization of compositions (Letunic and Bork, 2016). The ‘nestedness metric based on overlap and decreasing fill’ (Almeida‐Neto and Ulrich, 2011) was used to calculate nestedness among habitats as implemented in vegan. For this, samples were pooled per habitat and the pooled data were rarefied to the same number of reads per habitat.

Due to heterogeneity in our dataset, we used a machine learning technique (Random Forest) as implemented in *RandomForest* package (Liaw and Wiener, 2002) to determine whether the relative abundance of a given taxon can be predicted based on the effect of habitat and geography. In addition, indicator genera for each habitat were identified using a two‐way indicator species analysis following multiple testing correction, as implemented in *labdsv* package (https://cran.r-project.org/package=labdsv).

## Author contributions

M.B. and F.H. conceived the project; M.B., F.H., T.N., S.G. and P.B. designed the study; F.H. supervised bioinformatics; L.P.C. and P.F. organized the data, M.B., F.H. and C.F. performed data analysis; M.B. prepared the first draft and wrote the manuscript with F.H. and T.N.; All authors provided feedback and helped shape the manuscript.

## Supporting information


**Fig. S1.** Distribution of the metagenomic samples used in this study. Colours and size of symbols correspond to habitat types and number of samples, as indicated in the legend.

**Fig. S2.** Cross‐habitat differences in relative abundance of fungal classes.

**Fig. S3.** Cross‐habitat differences in relative abundance of fungal growth forms.

**Fig. S4.** Cross‐habitat differences in relative abundance of fungal functional guilds.

**Fig. S5.** Cross‐habitat differences in relative abundance of bacterial classes.

**Fig. S6.** The cross‐habitat canonical discriminant analysis of the composition of the top 20 bacterial and fungal classes associating most strongly to habitat types.

**Fig. S7.** Conceptual model showing patterns of fungal and bacterial diversity in relation to habitat type and gradients of associated factors that are proposed to be driving overall diversity patterns. The patterns of diversity of fungi appears to be associated with more spatially and temporally heterogeneous habitats with more available niches with higher and wider C:nutrient values and greater connectivity with other habitats, whereas bacteria show less discernible patterns with these factors, instead showing multiple diversity hotspots across a wide range of habitats, hinting that other more specific factors such as pH as the most important factor driving bacterial diversity, which fungi are less sensitive to.

**Fig. S8.** Soil as a main potential source for fungi but not bacteria in other habitats. The figure shows the occurrences (shaded cells) of top 100 most abundant bacterial and fungal genera across habitats, ordered according to the nestedness measure based on overlap and decreasing fill (NODF). NODF 0 and 100 indicate total randomness and perfect nestedness respectively. To minimize the effect of heterogeneous sequencing depths, data were pooled per habitat and rarefied to the same level across habitats. Text colours represent environmental, external host and internal host habitats, as indicated in the right panel.

**Fig. S9.** Boxplots of relative abundance of CAZyme substrates across three habitat clusters shown in Fig. 2. The boxplot uses only the median value for each category for the 25 habitats, the first p‐value refers to n = 25, to account for sample size differences between single habitats (e.g. human gut contained many more samples than Marine). The second p‐value (in parenthesis) indicates the p‐value across 13,452 samples and their distribution in three habitat clusters.

**Table S1.** Samples used in this study. The table presents a list of sample names, their sequencing and mapping statistics.

**Table S2.** Spearman correlations between CAZyme gene categories and the B/F ratio. Correlations were not done by discriminating on biomes.

**Table S3.** List of indicator bacterial and fungal genera associated with different biomes.

## Data Availability

All metagenomics sequences have been deposited in the European Bioinformatics Institute Sequence Read Archive database.

## References

[emi15314-bib-0001] Allison, S.D. , Lu, Y. , Weihe, C. , Goulden, M.L. , Martiny, A.C. , Treseder, K.K. , and Martiny, J.B. (2013) Microbial abundance and composition influence litter decomposition response to environmental change. Ecology 94: 714–725. 23687897 10.1890/12-1243.1

[emi15314-bib-0002] Almeida‐Neto, M. , and Ulrich, W. (2011) A straightforward computational approach for measuring nestedness using quantitative matrices. Environ Model Software 26: 173–178.

[emi15314-bib-0003] Averill, C. , and Hawkes, C.V. (2016) Ectomycorrhizal fungi slow soil carbon cycling. Ecol Lett 19: 937–947. 27335203 10.1111/ele.12631

[emi15314-bib-0004] Bahram, M. , Hildebrand, F. , Forslund, S.K. , Anderson, J.L. , Soudzilovskaia, N.A. , Bodegom, P.M. , *et al* (2018) Structure and function of the global topsoil microbiome. Nature 560: 233–237. 30069051 10.1038/s41586-018-0386-6

[emi15314-bib-0005] Bahram, M. , Netherway, T. , Hildebrand, F. , Pritsch, K. , Drenkhan, R. , Loit, K. , *et al* (2020) Plant nutrient‐acquisition strategies drive topsoil microbiome structure and function. In New Phylologist, Vol. 227, pp. 1189–1199. 10.1111/nph.16598 32279325

[emi15314-bib-0006] Bar‐On, Y.M. , Phillips, R. , and Milo, R. (2018) The biomass distribution on Earth. Proc Natl Acad Sci 115: 6506–6511. 29784790 10.1073/pnas.1711842115 PMC6016768

[emi15314-bib-0007] Berendsen, R.L. , Pieterse, C.M. , and Bakker, P.A. (2012) The rhizosphere microbiome and plant health. Trends Plant Sci 17: 478–486. 22564542 10.1016/j.tplants.2012.04.001

[emi15314-bib-0008] Boer, W. de, Folman, L.B. , Summerbell, R.C. , and Boddy, L. (2005) Living in a fungal world: impact of fungi on soil bacterial niche development. FEMS Microbiol Rev 29: 795–811, 4. 16102603 10.1016/j.femsre.2004.11.005

[emi15314-bib-0009] Buchfink, B. , Xie, C. , and Huson, D.H. (2014) Fast and sensitive protein alignment using DIAMOND. Nat Methods 12: 59–60. 25402007 10.1038/nmeth.3176

[emi15314-bib-0010] Bugg, T.D. , Ahmad, M. , Hardiman, E.M. , and Singh, R. (2011) The emerging role for bacteria in lignin degradation and bio‐product formation. Curr Opin Biotechnol 22: 394–400. 21071202 10.1016/j.copbio.2010.10.009

[emi15314-bib-0011] Cantarel, B.L. , Coutinho, P.M. , Rancurel, C. , Bernard, T. , Lombard, V. , and Henrissat, B. (2009) The Carbohydrate‐Active EnZymes database (CAZy): an expert resource for Glycogenomics. Nucleic Acids Res 37: D233–D238. 18838391 10.1093/nar/gkn663 PMC2686590

[emi15314-bib-0012] Crous, P.W. , Summerell, B.A. , Carnegie, A.J. , Wingfield, M.J. , Hunter, G.C. , Burgess, T.I. , *et al* (2009) Unravelling Mycosphaerella: do you believe in genera? Persoonia 23: 99–118. 20198164 10.3767/003158509X479487 PMC2802725

[emi15314-bib-0013] Crowther, T.W. , van den Hoogen, J. , Wan, J. , Mayes, M.A. , Keiser, A.D. , Mo, L. , *et al* (2019) The global soil community and its influence on biogeochemistry. Science 365: eaav0550. 31439761 10.1126/science.aav0550

[emi15314-bib-0014] de Boer, W. (2017) Upscaling of fungal–bacterial interactions: from the lab to the field. Curr Opin Microbiol 37: 35–41. 28437664 10.1016/j.mib.2017.03.007

[emi15314-bib-0015] de Vries, F.T. , and Caruso, T. (2016) Eating from the same plate? Revisiting the role of labile carbon inputs in the soil food web. Soil Biol Biochem 102: 4–9. 27812227 10.1016/j.soilbio.2016.06.023 PMC5061327

[emi15314-bib-0016] Delgado‐Baquerizo, M. , Oliverio, A.M. , Brewer, T.E. , Benavent‐González, A. , Eldridge, D.J. , Bardgett, R.D. , *et al* (2018) A global atlas of the dominant bacteria found in soil. Science 359: 320–325. 29348236 10.1126/science.aap9516

[emi15314-bib-0017] Deveau, A. , Bonito, G. , Uehling, J. , Paoletti, M. , Becker, M. , Bindschedler, S. , *et al* (2018) Bacterial‐fungal interactions: ecology, mechanisms and challenges. FEMS Microbiol Rev 42: 335–352. 29471481 10.1093/femsre/fuy008

[emi15314-bib-0018] Diao, Y.‐Z. , Chen, Q. , Jiang, X.‐Z. , Houbraken, J. , Barbosa, R.N. , Cai, L. , and Wu, W.‐P. (2019) Penicillium section Lanata‐divaricata from acidic soil. Cladistics 35: 514–549. 10.1111/cla.12365 34633696

[emi15314-bib-0019] Falkowski, P.G. , Fenchel, T. , and Delong, E.F. (2008) The microbial engines that drive Earth's biogeochemical cycles. Science 320: 1034–1039. 18497287 10.1126/science.1153213

[emi15314-bib-0020] Fisher, M.C. , Henk, D.A. , Briggs, C.J. , Brownstein, J.S. , Madoff, L.C. , McCraw, S.L. , and Gurr, S.J. (2012) Emerging fungal threats to animal, plant and ecosystem health. Nature 484: 186–194. 22498624 10.1038/nature10947 PMC3821985

[emi15314-bib-0021] Frey‐Klett, P. , Burlinson, P. , Deveau, A. , Barret, M. , Tarkka, M. , and Sarniguet, A. (2011) Bacterial‐fungal interactions: hyphens between agricultural, clinical, environmental, and food microbiologists. Microbiol Mol Biol Rev 75: 583–609. 22126995 10.1128/MMBR.00020-11 PMC3232736

[emi15314-bib-0022] Friendly, M. , Fox, J. , and Friendly, M.M . (2017) Package ‘candisc.’ https://CRAN.R-project.org/package=candisc.

[emi15314-bib-0023] Galili, T. (2015) Dendextend: an R package for visualizing, adjusting and comparing trees of hierarchical clustering. Bioinformatics 31: 3718–3720. 26209431 10.1093/bioinformatics/btv428 PMC4817050

[emi15314-bib-0024] Grice, E.A. , and Segre, J.A. (2011) The skin microbiome. Nat Rev Microbiol 9: 244–253. 21407241 10.1038/nrmicro2537 PMC3535073

[emi15314-bib-0025] Handelsman, J. (2004) Metagenomics: application of genomics to uncultured microorganisms. Microbiol Mol Biol Rev 68: 669–685. 15590779 10.1128/MMBR.68.4.669-685.2004 PMC539003

[emi15314-bib-0026] Hannula, S.E. , Zhu, F. , Heinen, R. , and Bezemer, T.M. (2019) Foliar‐feeding insects acquire microbiomes from the soil rather than the host plant. Nat Commun 10: 1–9. 30890706 10.1038/s41467-019-09284-w PMC6425034

[emi15314-bib-0027] Hauswedell, H. , Singer, J. , and Reinert, K. (2014) Lambda: the local aligner for massive biological data. Bioinformatics 30: i349–i355. 25161219 10.1093/bioinformatics/btu439 PMC4147892

[emi15314-bib-0028] Hildebrand, F. , Moitinho‐Silva, L. , Blasche, S. , Jahn, M.T. , Gossmann, T.I. , Huerta‐Cepas, J. , *et al* (2019) Antibiotics‐induced monodominance of a novel gut bacterial order. In Gut, Vol. 68, pp. 1781–1790. 30658995 10.1136/gutjnl-2018-317715 PMC6839795

[emi15314-bib-0029] Hildebrand, F. , Tadeo, R. , Voigt, A.Y. , Bork, P. , and Raes, J. (2014) LotuS: an efficient and user‐friendly OTU processing pipeline. Microbiome 2: 30. 27367037 10.1186/2049-2618-2-30 PMC4179863

[emi15314-bib-0030] Hoffmann, C. , Dollive, S. , Grunberg, S. , Chen, J. , Li, H. , Wu, G.D. , *et al* (2013) Archaea and fungi of the human gut microbiome: correlations with diet and bacterial residents. PLOS ONE 8: e66019. 23799070 10.1371/journal.pone.0066019 PMC3684604

[emi15314-bib-0031] Huang, M. , and Hull, C.M. (2017) Sporulation: how to survive on planet Earth (and beyond). Curr Genet 63: 831–838. 28421279 10.1007/s00294-017-0694-7 PMC5647196

[emi15314-bib-0032] Huerta‐Cepas, J. , Szklarczyk, D. , Forslund, K. , Cook, H. , Heller, D. , Walter, M.C. , *et al* (2015) eggNOG 4.5: a hierarchical orthology framework with improved functional annotations for eukaryotic, prokaryotic and viral sequences. Nucleic Acids Res 44: D286–D293. 26582926 10.1093/nar/gkv1248 PMC4702882

[emi15314-bib-0033] Hugenholtz, P. , and Tyson, G.W. (2008) Metagenomics. Nature 455: 481–483. 18818648 10.1038/455481a

[emi15314-bib-0034] Keiblinger, K.M. , Hall, E.K. , Wanek, W. , Szukics, U. , Hämmerle, I. , Ellersdorfer, G. , *et al* (2010) The effect of resource quantity and resource stoichiometry on microbial carbon‐use‐efficiency. FEMS Microbiol Ecol 73: 430–440. 20550579 10.1111/j.1574-6941.2010.00912.x

[emi15314-bib-0035] Kopylova, E. , Noé, L. , and Touzet, H. (2012) SortMeRNA: fast and accurate filtering of ribosomal RNAs in metatranscriptomic data. Bioinformatics 28: 3211–3217. 23071270 10.1093/bioinformatics/bts611

[emi15314-bib-0036] Küngas, K. , Bahram, M. , and Põldmaa, K. (2020) Host tree organ is the primary driver of endophytic fungal community structure in a hemiboreal forest. FEMS Microbiol Ecol 96: fiz199. 31825516 10.1093/femsec/fiz199

[emi15314-bib-0037] Letunic, I. , and Bork, P. (2016) Interactive tree of life (iTOL) v3: an online tool for the display and annotation of phylogenetic and other trees. Nucleic Acids Res 44: W242–W245. 27095192 10.1093/nar/gkw290 PMC4987883

[emi15314-bib-0038] Liaw, A. , and Wiener, M. (2002) Classification and regression by randomForest. R News 2: 18–22.

[emi15314-bib-0039] Louca, S. , Parfrey, L.W. , and Doebeli, M. (2016) Decoupling function and taxonomy in the global ocean microbiome. Science 353: 1272–1277. 27634532 10.1126/science.aaf4507

[emi15314-bib-0040] Lutzoni, F. , Nowak, M.D. , Alfaro, M.E. , Reeb, V. , Miadlikowska, J. , Krug, M. , *et al* (2018) Contemporaneous radiations of fungi and plants linked to symbiosis. Nat Commun 9: 5451. 30575731 10.1038/s41467-018-07849-9 PMC6303338

[emi15314-bib-0041] Lynch, M. , and Walsh, B. (2007) The Origins of Genome Architecture. Sunderland, MA: Sinauer Associates.

[emi15314-bib-0042] Magoč, T. , and Salzberg, S.L. (2011) FLASH: fast length adjustment of short reads to improve genome assemblies. Bioinformatics 27: 2957–2963. 21903629 10.1093/bioinformatics/btr507 PMC3198573

[emi15314-bib-0043] Mendes, R. , Kruijt, M. , de Bruijn, I. , Dekkers, E. , van der Voort, M. , Schneider, J.H.M. , *et al* (2011) Deciphering the rhizosphere microbiome for disease‐suppressive Bacteria. Science 332: 1097–1100. 21551032 10.1126/science.1203980

[emi15314-bib-0044] Naranjo‐Ortiz, M.A. , and Gabaldón, T. (2019) Fungal evolution: major ecological adaptations and evolutionary transitions. Biol Rev 94: 1443–1476. 31021528 10.1111/brv.12510 PMC6850671

[emi15314-bib-0045] Nguyen, N.H. , Song, Z. , Bates, S.T. , Branco, S. , Tedersoo, L. , Menke, J. , *et al* (2016) FUNGuild: an open annotation tool for parsing fungal community datasets by ecological guild. Fungal Ecol 20: 241–248.

[emi15314-bib-0046] Oksanen, J. , Kindt, R. , Legendre, P. , O'Hara, B. , Stevens, M.H.H. , Oksanen, M.J. , and Suggests, M. (2007) The vegan package. Community Ecol Package 10: 631–637.

[emi15314-bib-0047] Pent, M. , Põldmaa, K. , and Bahram, M. (2017) Bacterial communities in boreal forest mushrooms are shaped both by soil parameters and host identity. Front Microbiol 8: 836. 28539921 10.3389/fmicb.2017.00836 PMC5423949

[emi15314-bib-0048] Phelan, V.V. , Liu, W.‐T. , Pogliano, K. , and Dorrestein, P.C. (2012) Microbial metabolic exchange—the chemotype‐to‐phenotype link. Nat Chem Biol 8: 26–35. 10.1038/nchembio.739 PMC3869239 22173357

[emi15314-bib-0049] Quast, C. , Pruesse, E. , Yilmaz, P. , Gerken, J. , Schweer, T. , Yarza, P. , *et al* (2012) The SILVA ribosomal RNA gene database project: improved data processing and web‐based tools. Nucleic Acids Res 41: D590–D596. 23193283 10.1093/nar/gks1219 PMC3531112

[emi15314-bib-0050] Quince, C. , Walker, A.W. , Simpson, J.T. , Loman, N.J. , and Segata, N. (2017) Shotgun metagenomics, from sampling to analysis. Nat Biotechnol 35: 833. 28898207 10.1038/nbt.3935

[emi15314-bib-0051] Rohart, F. , Gautier, B. , Singh, A. , and Lê Cao, K.‐A. (2017) mixOmics: an R package for 'omics feature selection and multiple data integration. PLoS Comput Biol 13: e1005752. 29099853 10.1371/journal.pcbi.1005752 PMC5687754

[emi15314-bib-0052] Rosling, A. , Cox, F. , Cruz‐Martinez, K. , Ihrmark, K. , Grelet, G.‐A. , Lindahl, B.D. , *et al* (2011) Archaeorhizomycetes: unearthing an ancient class of ubiquitous soil fungi. Science 333: 876–879. 21836015 10.1126/science.1206958

[emi15314-bib-0053] Schmidt, T.S. , Raes, J. , and Bork, P. (2018) The human gut microbiome: from association to modulation. Cell 172: 1198–1215. 29522742 10.1016/j.cell.2018.02.044

[emi15314-bib-0054] Sokol, H. , Leducq, V. , Aschard, H. , Pham, H.‐P. , Jegou, S. , Landman, C. , *et al* (2017) Fungal microbiota dysbiosis in IBD. Gut 66: 1039–1048. 26843508 10.1136/gutjnl-2015-310746 PMC5532459

[emi15314-bib-0055] Strickland, M.S. , and Rousk, J. (2010) Considering fungal: bacterial dominance in soils–methods, controls, and ecosystem implications. Soil Biol Biochem 42: 1385–1395.

[emi15314-bib-0056] Tedersoo, L. , Anslan, S. , Bahram, M. , Põlme, S. , Riit, T. , Liiv, I. , *et al* (2015) Shotgun metagenomes and multiple primer pair‐barcode combinations of amplicons reveal biases in metabarcoding analyses of fungi. MycoKeys 10: 1–43.

[emi15314-bib-0057] Tedersoo, L. , Bahram, M. , Polme, S. , Koljalg, U. , Yorou, N.S. , Wijesundera, R. , *et al* (2014) Global diversity and geography of soil fungi. Science 346: 1256688–1256688. 25430773 10.1126/science.1256688

[emi15314-bib-0058] Tedersoo, L. , Sánchez‐Ramírez, S. , Koljalg, U. , Bahram, M. , Döring, M. , Schigel, D. , *et al* (2018) High‐level classification of the Fungi and a tool for evolutionary ecological analyses. Fungal Divers 90: 135–159.

[emi15314-bib-0059] Thompson, L.R. , Sanders, J.G. , McDonald, D. , Amir, A. , Ladau, J. , *et al* (2017) A communal catalogue reveals Earth's multiscale microbial diversity. Nature 551: 457–463. 29088705 10.1038/nature24621 PMC6192678

[emi15314-bib-0060] Whiteside, M.D. , Werner, G.D. , Caldas, V.E. , van't Padje, A. , Dupin, S.E. , Elbers, B. , *et al* (2019) Mycorrhizal fungi respond to resource inequality by moving phosphorus from rich to poor patches across networks. In Curr Biol, Vol. 29, pp. 2043–2050.e8. 31178314 10.1016/j.cub.2019.04.061 PMC6584331

[emi15314-bib-0061] Wilhelm, R.C. , Singh, R. , Eltis, L.D. , and Mohn, W.W. (2019) Bacterial contributions to delignification and lignocellulose degradation in forest soils with metagenomic and quantitative stable isotope probing. ISME J 13: 413–429. 30258172 10.1038/s41396-018-0279-6 PMC6331573

[emi15314-bib-0062] Worrich, A. , Stryhanyuk, H. , Musat, N. , König, S. , Banitz, T. , Centler, F. , *et al* (2017) Mycelium‐mediated transfer of water and nutrients stimulates bacterial activity in dry and oligotrophic environments. Nat Commun 8: 15472. 28589950 10.1038/ncomms15472 PMC5467244

[emi15314-bib-0063] Yarza, P. , Yilmaz, P. , Pruesse, E. , Glöckner, F.O. , Ludwig, W. , Schleifer, K.‐H. , *et al* (2014) Uniting the classification of cultured and uncultured bacteria and archaea using 16S rRNA gene sequences. Nat Rev Microbiol 12: 635–645. 25118885 10.1038/nrmicro3330

[emi15314-bib-0064] Žifčáková, L. , Větrovský, T. , Lombard, V. , Henrissat, B. , Howe, A. , and Baldrian, P. (2017) Feed in summer, rest in winter: microbial carbon utilization in forest topsoil. Microbiome 5: 122. 28923122 10.1186/s40168-017-0340-0 PMC5604414

